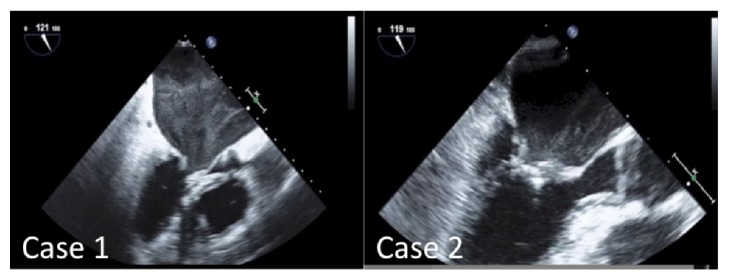# Repeat Minimally Invasive Mitral Valve Replacement for Recurrent Mitral Stenosis after OMC in Patients Who Decline Blood Product Transfusion for Religious Reasons

**DOI:** 10.1155/2015/239197

**Published:** 2015-11-05

**Authors:** Yujiro Ito, Yoshitsugu Nakamura, Osamu Tagusari, Shigehiko Yoshida

**Affiliations:** ^1^Department of Cardiovascular Surgery, Chibanishi General Hospital, 107-1 Kanegasaku, Matsudo, Chiba 270-2251, Japan; ^2^Department of Cardiovascular Surgery, Omori Red Cross Hospital, 4-30-1 Chuo, Ota-ku, Tokyo 143-8527, Japan; ^3^Department of Cardiovascular Surgery, IMS Katsushika Heart Center, 3-30-1 Horikiri, Katsushika-ku, Tokyo 124-0006, Japan

## Abstract

Cardiac surgery for Jehovah's Witness (JW) patients is considered to be high risk because of patients' refusal to receive blood transfusion. We report a successful mitral valve replacement for recurrent mitral stenosis after OMC with minimally invasive right thoracotomy, without any transfusion of allogeneic blood or blood products. This minimally invasive mitral valve replacement through right thoracotomy was an excellent approach for JW patients.

## 1. Introduction

The invasive nature of cardiac surgery and the use of CPB may lead to blood loss that may require several blood transfusions during the procedure. Therefore, cardiac surgery for a Jehovah's Witness patient is considered to be high risk because of the patient's refusal to receive blood transfusion [1]. Less postoperative bleeding, low need for blood transfusion, and absence of sternal wound infection are the main advantages of minimally invasive cardiac surgery (MICS).

We report a successful second complex cardiac operation following an open mitral commissurotomy without any transfusion of allogeneic blood or blood products in two female Jehovah's Witness patients with MICS.

## 2. Case Presentation

### 2.1. Case 1

A 71-year-old female was referred to our institution two years ago, with dyspnea due to reoccurrence of mitral stenosis. She had a past medical history of rheumatic fever and mitral stenosis and received open mitral commissurotomy (OMC) 28 years ago. She was also a believer of Jehovah's Witnesses (JW). Echocardiography revealed severe mitral stenosis, but because surgical treatment was considered to be high risk, she had been followed up medically for several years. However, there had been a gradual exacerbation of her symptoms compatible with NYHA class III during follow-up. Finally, her surgical treatment was scheduled.

**Figure 1:**
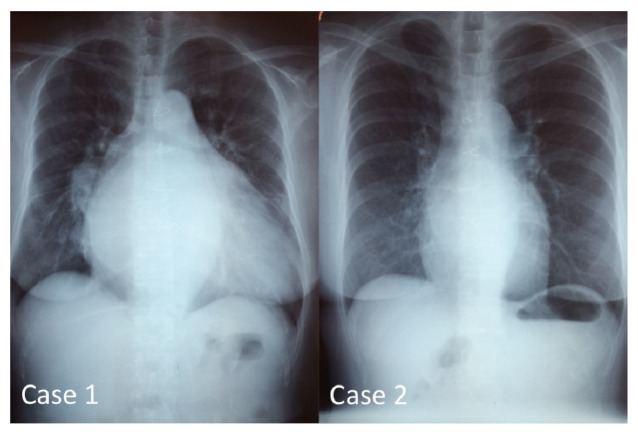


Chest X-ray showed cardiomegaly and protrusion of the right aortic arches (see Figure 1).

**Figure 2:**
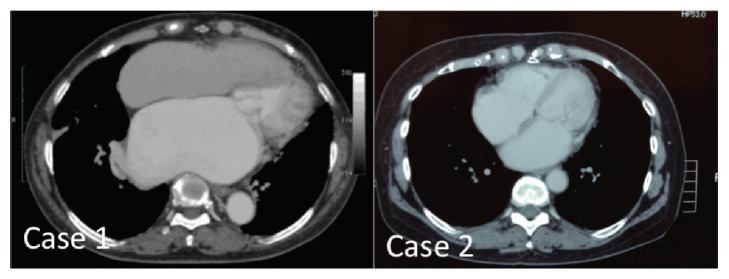


CT showed enlarged left atrium accompanied by displaced left ventricle (see Figure 2).

Echocardiography revealed severe mitral stenosis with MVA of 0.92 cm^2^, with mild mitral regurgitation. Left atrium was enlarged to 65 mm. LV function was well preserved and LVDd/Ds was measured to be 51/33, with an EF of 64%. Intraoperative TEE showed enlarged left atrium with smoke-like echo and thickening and doming motion of the mitral leaflet (see Figure 3).

There was moderate to severe tricuspid regurgitation; therefore, we concomitantly planned a tricuspid annuloplasty.

Erythropoietin (EPO) was started until the hemoglobin reached at least 14 mg/dL, and the preoperative hemoglobin level was 16.0 mg/dL.

To minimize bleeding and possible injury from a resternotomy, we decided to perform mitral valve replacement with mini-right thoracotomy.

The patient underwent a minimally invasive right thoracotomy, 8 cm in length, through the right 4th intercostal space. Cardiopulmonary bypass was established through direct cannulation of right femoral artery and vein, and percutaneous cannulation of right jugular vein with vacuum assisted venous return. The ascending aorta was carefully dissected and then an aortic cross clamp was placed with flexible transthoracic-aortic clamp through the main incision. Myocardial protection was given every 20 min using intermittent antegrade cold blood cardioplegia through an aortic root cannula. The right side of the left atrium was opened and mitral valve replacement was performed using a 23 mm Carpentier-Edwards PERIMOUNT Magna Mitral Ease (Edwards; Lifesciences Inc., Irvine, CA, USA). After closing the left atrium, and snaring down the vena cava, right atrium was opened longitudinally, and tricuspid annuloplasty was performed with De Vega method. Subsequently, the aortic cross clamp was released. Cardiac arrest time was 107 min. The surgery time was 350 min.

The hemoglobin level at the end of the procedure was 16.7 g/dL and intraoperative blood loss was 300 mL. The patient had a reexpansion pulmonary edema postoperatively but was extubated on the third postoperative day. The lowest hemoglobin during the postoperative course was 13.4 g/dL on the third postoperative day. The patient was discharged from the hospital on the 10th postoperative day with a hemoglobin level of 13.8 g/dL. Postoperative echocardiography revealed MVA of 2.10 cm^2^.

### 2.2. Case 2

A 61-year-old female was referred to our institution for dyspnea due to recurrent mitral stenosis. She had a past medical history of mitral stenosis and received open mitral commissurotomy (OMC) 28 years ago. She started to feel palpitation and shortness of breath 8 years ago, and recently her symptom was compatible with NYHA class III. She was also a believer of Jehovah's Witnesses. Surgical treatment was considered to be high risk, but because her symptom was severe, we decided to schedule surgical treatment.

Echocardiography revealed severe mitral stenosis with MVA of 1.19 cm^2^, with trace mitral regurgitation. Left atrium was enlarged to be 47 mm. LV function was well preserved and LVDd/Ds was measured to be 42/27, with an EF of 65%. She also had chronic atrial fibrillation, so maze procedure was also planned.

The patient underwent a minimally invasive right thoracotomy, 8 cm in length, through the right 4th intercostal space. Cardiopulmonary bypass was established with the same manner as with case 1. The left atrium was opened through an incision parallel to the interatrial groove. Maze procedure of the left atrium was performed with cryoablation. Subsequently, mitral valve replacement was performed with 25 mm St. Jude Medical mechanical valve. The left atrium was closed and the aortic cross clamp was released. Cardiac arrest time was 110 min. The surgery time was 280 min.

The level of hemoglobin at the end of the procedure was 11.5 g/dL and intraoperative blood loss was 200 mL. The patient was extubated on the first postoperative day. The lowest hemoglobin during the postoperative course was 9.8 g/dL on the second postoperative day. The patient was discharged from the hospital on the 6th postoperative day with a hemoglobin level of 11.7 g/dL. Postoperative echocardiography revealed MVA of 2.50 cm^2^.

## 3. Discussion

The invasive nature of cardiac surgery and the use of CPB may lead to blood loss that may require several blood transfusions during the procedure. On the other hand, the techniques of blood conservation have improved greatly and have become rather systematized, together with an overall decrease in operative risk of cardiac surgery. Emmert et al. [2] reported the safety and feasibility of open-heart surgery in JW patients and highlighted that patient blood management led to excellent short-term and long-term clinical outcomes. Preoperative intravenous iron supplementation and erythropoietin were recommended to raise the hemoglobin level. Body temperature was continuously monitored and hypothermia <35°C was avoided using warming systems to reduce blood loss due to coagulopathy. In addition, to maintain an intact circuit to the patient, a continuous connection was guaranteed from the Cell Saver washed blood bag and connected to the patient's intravenous line [2, 3]. In both the cases, erythropoietin (EPO) was given preoperatively until the hemoglobin reached at least 14 mg/dL. We use Cell Saver routinely for cardiac surgeries; therefore, it was used in the two cases as well. Postoperative intravenous and oral iron supplement were given.

Falk et al. [4] indicated that, in patients with mitral valve disease, minimally invasive surgery could be an alternative to conventional mitral valve surgery with comparable short-term and long-term mortality and in-hospital morbidity. It has some disadvantages of prolonged cross clamp time, CPB time, and procedure time, but reduced sternal complications, transfusions, duration of ventilation, and intensive care unit and hospital length of stay were strong advantages [5]. Moreover, Murzi et al. [6] reported that minimally invasive mitral valve reoperation can also be performed with an operative mortality similar to standard sternotomy approach. Minimally invasive reoperation had few conversion to sternotomy and less amount of bleeding and blood transfusion compared with sternotomy [7]. In both the cases, we performed several extra procedures for blood conservation along with our general MICS procedure. We created a larger incision to insert venting tubes and aortic cross clamps from the same incision. Usually in MICS, venting tubes and aortic cross clamps are inserted from different intercostal spaces because of a small incision, but this time we inserted them from the main incision to avoid extra bleeding from the chest walls. Moreover, we checked the bleeding from the chest wall using an endoscope before closing the incision.

The two patients in this study not only refused blood product transfusion, but also had previous cardiac surgeries, so careful blood management was required. From the advantages mentioned, minimally invasive surgery was suitable.

## Figures and Tables

**Figure 3:**